# Association between Liver Damage and Disease Progression Markers with Mortality Risk and Mechanical Ventilation in Hospitalized COVID-19 Patients: A Nationwide Retrospective SARSTer Study

**DOI:** 10.3390/v16101530

**Published:** 2024-09-27

**Authors:** Karol Żmudka, Jerzy Jaroszewicz, Dorota Zarębska-Michaluk, Magdalena Rogalska, Piotr Czupryna, Marta Rorat, Dorota Kozielewicz, Jadwiga Maciukajć, Sławomir Kiciak, Magdalena Krępa, Ewa Dutkiewicz, Michał Stojko, Aleksandra Spychał, Przemysław Ciechanowski, Beata Bolewska, Regina Podlasin, Robert Flisiak

**Affiliations:** 1 Department of Infectious Diseases and Hepatology, Medical University of Silesia, 40-635 Katowice, Polands83148@365.sum.edu.pl (M.S.); s81359@365.sum.edu.pl (A.S.); 2 Department of Infectious Diseases and Allergology, Jan Kochanowski University, 25-317 Kielce, Poland; 3 Department of Infectious Diseases and Hepatology, Medical University of Bialystok, 15-540 Bialystok, Poland; pmagdar@gmail.com (M.R.); robert.flisiak1@gmail.com (R.F.); 4 Department of Infectious Diseases and Neuroinfections, Medical University of Bialystok, 15-540 Bialystok, Poland; 5 Department of Social Sciences and Infectious Diseases, Medical Faculty, Wroclaw University of Science and Technology, 50-470 Wroclaw, Poland; 6 Department of Infectious Diseases and Hepatology, Faculty of Medicine, Collegium Medicum in Bydgoszcz, Nicolaus Copernicus University, 85-030 Bydgoszcz, Poland; 7 Department of Infectious Diseases, District Healthcare Center, 27-200 Starachowice, Poland; 8 Independent Voivodeship Hospital “Jana Bożego” in Lublin, 20-400 Lublin, Poland; 9 Szpital Powiatowy w Mielcu, 39-300 Mielec, Poland; 10 Department of Pediatrics and Infectious Diseases, Regional Hospital in Szczecin, 71-252 Szczecin, Poland; 11 Department of Infectious Diseases, Poznań University of Medical Sciences, 61-285 Poznan, Poland; 12 IV-th Department, Hospital for Infectious Diseases, 01-201 Warsaw, Poland; podlasin@zakazny.pl; 13 Department of Infectious Diseases, Collegium Medicum, Cardinal Stefan Wyszynski University in Warsaw, 01-815 Warsaw, Poland

**Keywords:** SARS-CoV-2, COVID-19, liver, liver fibrosis, alanine aminotransferase, aspartate aminotransferase, liver function tests, lactate dehydrogenases, mortality, respiration, artificial

## Abstract

(1) Background: Liver damage is an important component of acute COVID-19, and the advancement of preexisting liver disease is associated with a worse prognosis; (2) Methods: A nationwide retrospective study including 7444 patients aimed to evaluate levels of selected markers of liver damage and disease advancement and their association with mortality and mechanical ventilation (MV); (3) Results: Elevation of the following markers in multivariate models were associated with increased odds of mortality: Alanine transaminase (ALT), aspartate transaminase (AST), gamma-glutamyltransferase (GGT), lactate dehydrogenase (LDH), fibrosis-4 score (FIB-4), AST-to-platelet ratio index (APRI), and decreased levels of platelet count (PLT). Elevated levels of AST, LDH, APRI, FIB-4, and the AST/ALT ratio and decreased levels of PLT were associated with increased odds of MV in multivariate models. The best predictive accuracy against mortality was achieved with FIB-4 with AUC = 0.733 (95% CI, 0.718–0.749) at the optimal cut-off point of 2.764, while against MV was achieved with LDH with AUC = 0.753 (95% CI, 0.727–0.778) at the optimal cut-off point of 449.5 IU/L. (4) Conclusions: Our study confirms that the advancement of liver damage contributes to a worse prognosis in COVID-19 patients. Markers for liver damage and the advancement of liver disease can provide predictive value in clinical practice among COVID-19 patients.

## 1. Introduction

Severe acute respiratory syndrome coronavirus 2 (SARS-CoV-2) is a cause of coronavirus disease 2019 (COVID-19) that led to a global pandemic [1]. Initially, the respiratory tract was considered the primary target of SARS-CoV-2 [2]. However, it has been noticed that in many patients, the liver is also affected. The angiotensin-converting enzyme 2 (ACE2), which is the main host receptor to which the spike protein of the virus binds, is also expressed in the liver, especially in cholangiocytes. Liver injury caused by SARS-CoV-2 is likely caused by multiple factors, such as the direct cytopathic effect of the virus, an exaggerated systematic immune response due to a cytokine storm, vascular damage, coagulopathy, and drug-induced injury [2,3]. Several biomarkers are used in the clinical diagnostic process to help in identifying liver injury.

Alanine aminotransferase (ALT) and aspartate aminotransferase (AST) take part in gluconeogenesis in the liver and are released into the blood in hepatocytic injury. In clinical diagnostics to interpret levels of AST and ALT, the AST/ALT ratio, also known as the DeRitis ratio, is often used. Gamma glutamyl transferase (GGT) is present in many organs, including the liver, and its elevated levels, apart from liver injury, can be caused by drugs, alcohol use, myocardial infarction, chronic obstructive pulmonary disease, pancreatic disease, or renal failure [4]. L-lactate dehydrogenases (LDH) are a family of 2-hydroxy acid oxidoreductases that interconvert pyruvate to lactate. LDH is found in skeletal muscles, cardiac muscles, and liver [5]. Elevated levels of LDH in COVID-19 patients have been observed in those with abnormal liver function as well as in those who died from respiratory failure. Increased levels of LDH may also be associated with injury of the liver and heart in COVID-19, as ACE2 is widely expressed in cardiac blood vessels. Injury of the liver in COVID-19 patients is also linked to the hepatotoxicity of the drugs involved in the treatment, which could explain elevated levels of LDH [6]. An aspect of platelet activity remains an interest in COVID-19 due to thrombotic complications in this disease. Thrombocytopenia has been reported in hospitalized COVID-19 patients as an indicator of poor clinical outcomes. Moreover, post-mortem liver biopsies of deceased patients demonstrated portal or sinusoidal vascular thrombosis in at least 50% of patients [7,8]. Platelet count is also a part of the fibrosis index based on four factors (FIB-4), which includes levels of AST and ALT as well. It is calculated by using the following formula: (age [years] × AST [U/L])/(PLT [10^9^/L] × (ALT [U/L])1/2), and it has been proven to be accurate in classifying different stages of liver fibrosis in patients with viral hepatitis and non-alcoholic fatty liver disease (NAFLD). FIB-4 seems to perform better than the AST-to-platelet ratio index (APRI) in detecting liver fibrosis [9]. The APRI score contains AST and platelet scores, and it is calculated using [AST (U/L)/upper limit of normal × 100]/platelet (×10^9^/L) ratio]. Furthermore, it has been shown that a score below 1 in COVID-19 patients is associated with higher deaths and a lower rate of hospital discharges [10]. Our study aimed to evaluate the association between levels of selected biomarkers of liver injury and disease advancement with in-hospital mortality and the need for mechanical ventilation among hospitalized COVID-19 patients. We established that elevated levels of liver injury biomarkers, as well as elevated markers of liver damage advancement, were associated with increased odds of both mortality and the need for mechanical ventilation. Some of those markers can be used as good predictors of those events.

## 2. Materials and Methods

### 2.1. Database

The data for this study come from the SARSTer national database, which includes retrospective data of 13,636 COVID-19 patients hospitalised between 1 March 2020 and 15 May 2023 in 51 Polish centres. SARSTer is an ongoing national real-world experience study assessing treatment outcomes in patients with COVID-19 supported by the Polish Association of Epidemiologists and Infectious Diseases Physicians. More information about the SARSTer project has been described elsewhere [11]. From the cohort of 13,635 patients, we excluded those who lacked reported mortality data, data concerning need for mechanical ventilation, or had missing AST, ALT, or PLT levels and obtained a cohort of 8004 patients. From that cohort we excluded patients with extreme results (*n* = 560, 7%) and obtained a cohort of 7444, which was a subject of further analysis. Extreme results were trimmed from the database by removing 1st and 99th percentile results from each of the measured and analysed variables (ALT, AST, GGT, LDH, PLT). Patients were tested for COVID-19 through real-time reverse transcriptase–polymerase chain reaction (RT–PCR) or a COVID-19 rapid antigen test.

### 2.2. Analysed Parameters

The overall condition of each patient was assessed by clinical presentation and O_2_ saturation measured at admission and later stratified into 5 groups of COVID-19 disease severity—asymptomatic; symptomatic and stable patients with O_2_ saturation without oxygen >95%; symptomatic and unstable patients with O_2_ saturation without oxygen ≤95%; symptomatic unstable patients with SpO_2_ without oxygen ≤90%; and acute respiratory distress syndrome patients. In this study, we analysed demographic data of the patients, the presence of comorbid diseases, and levels of O_2_ saturation and selected available parameters in the database associated with liver function (ALT, AST, GGT, LDH levels, and PLT count), and scoring systems based on those data (FIB-4 index, APRI index, and ASL/ALT ratio). In the analysis of those factors, we focused on the predictors of in-hospital mortality and the need for mechanical ventilation.

### 2.3. Statistical Analysis

We report medians and interquartile ranges (IQRs) for continuous variables and the number of cases with proportions for categorical variables. Continuous variables were assessed using the Mann–Whitney U test, while categorical variables were analysed using the chi–square test. For all analyses, two-sided *p* values ≤ 0.05 were considered statistically significant. Logistic regression models were employed to determine the factors influencing the likelihood of in-hospital mortality and the requirement for mechanical ventilation. We present the crude odds ratio (OR) for univariate logistic regression models and adjusted OR for multivariate models. Multivariate models were adjusted for age, sex, BMI, O_2_ saturation at admission and presence of comorbidities: malignancy, hypertension, diabetes, ischaemic heart disease, and COPD. We present the OR with a confidence interval (CI) of 95%. We performed a receiver operating characteristic (ROC) curve analysis and the Youden index was utilised to determine the optimal cut-off point for predicting mortality or need for mechanical ventilation. The area under the curve (AUC) was calculated using the Delong method [12]. Additionally, to present time-to-event data related to mortality, we utilised the Kaplan–Meier estimator. Since time-to-event data for mechanical ventilation was unavailable, we did not conduct a similar analysis for this event. Lastly, we analysed a cohort of asymptomatic patients and reported the number and proportion of patients with abnormal results. All statistical analyses were performed using RStudio statistical software (2023.09.0+463).

## 3. Results

### 3.1. Population Characteristics

In the studied population, the median age of the patients was 65 years (IQR: 49–76), and around half of the population was male (52.7%). Around a quarter of the population (25.4%) was obese, with a median BMI equal to 27 kg/m^2^ (IQR: 23.7–30.8). Median hospitalisation time was 10 days (IQR: 7–14). When analysing patients’ condition at admission, asymptomatic patients accounted for 196 cases (2.6%), symptomatic and stable patients with O_2_ saturation without oxygen >95% accounted for 1986 cases (27%) of the studied population, symptomatic and unstable patients with O_2_ saturation without oxygen ≤95% accounted for 2437 patients (32.7%), 2635 (35.4%) were symptomatic unstable patients with SpO_2_ without oxygen ≤90%, and 73 (0.01%) were ARDS patients, while 117 (1.6%) patients were not classified in either of the categories. Overall median O_2_ saturation in the studied population equalled to 92% (IQR: 88–95). Moreover, when analysing the period of admission, 3930 (52.8%) patients were assessed as pre-Delta COVID-19, 1992 (26.8%) as Delta, and 1522 (20.4%) as Omicron. We present below the median levels of the analysed laboratory findings: ALT 29 (IQR: 19–47) IU/L, AST 39 (IQR: 28–58) IU/L, GGT 40 (IQR: 22–76) IU/L, LDH 334 (IQR: 244–464) IU/L, and PLT 192,000 (IQR: 145,000–255,000) 1/μL; as well as the calculated indexes based on those results: APRI 0.523 (IQR: 0.328–0.879), FIB-4 score 2.34 (IQR: 1.31–3.97), and AST/ALT ratio 1.34 (IQR: 1–1.84). Table 1 presents demographic, clinical, and laboratory characteristics categorised by survival status and the necessity for mechanical ventilation among hospitalised patients.

### 3.2. Univariate and Multivariate Logistic Regression Models

In univariate logistic regressions, elevated levels of AST, GGT, LDH, APRI, FIB-4, AST/ALT ratio, and a decreased count of PLT were associated with increased odds of in-hospital mortality, while elevated ALT was not significantly associated. Each marker was adjusted for age, sex, BMI, and O_2_ saturation at admission, and the presence of comorbidities: malignancy, hypertension, diabetes, ischaemic heart disease, and COPD. When adjusted, all factors were associated with increased odds of in-hospital mortality, with an exception to increased PLT count, which was associated with decreased odds of in-hospital mortality.

When analysing the factors associated with increased odds of requiring mechanical ventilation in the univariate model, ALT, AST, LDH, APRI, FIB-4, and the AST/ALT ratio were associated with increased odds of needing mechanical ventilation, while an elevated count of PLT was associated with decreased odds. However, in the multivariate model, only increased levels of AST, LDH, APRI, FIB-4, and the AST/ALT ratio were associated with increased odds of mechanical ventilation, while a PLT increase was associated with decreased odds. We present results of univariate and multivariate logistic regression models for the odds of in-hospital mortality in Table 2 and for the odds of mechanical ventilation in Table 3. Detailed information about each of the 16 multivariate logistic regression models is depicted in the Supplementary File.

### 3.3. ROC Analysis

A receiver-operator characteristic analysis revealed that FIB-4 presented the highest predictive value for mortality among all analysed factors (AUC 0.73 [95%CI: 0.72–0.75]) at the optimal cut-off point of 2.76, while the highest predictive value for mechanical ventilation was observed for LDH 0.75 (95%CI: [0.73–0.78]) at the optimal cut-off point of 449.5 IU/L. Detailed ROC analysis for mortality and need for mechanical ventilation is presented in Table 4 and Table 5, respectively.

**Figure 1:**
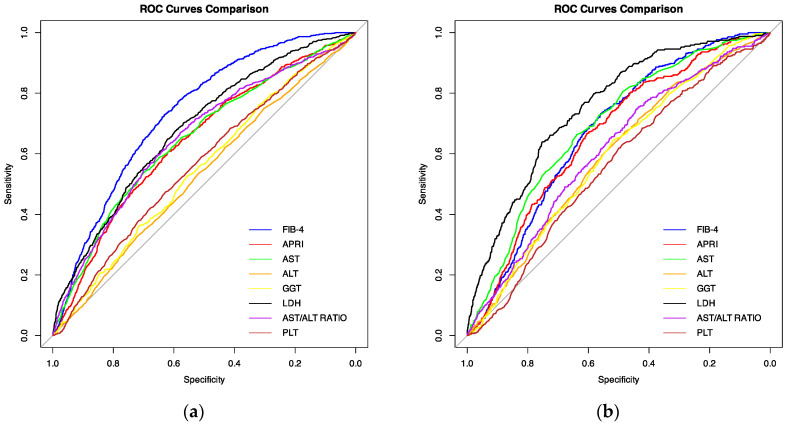
Comparison between receiver operator characteristic (ROC) curves of analysed biomarkers and indexes (ALT, alanine transaminase; AST, aspartate transaminase; GGT, gamma-glutamyltransferase; LDH, lactate dehydrogenase; PLT, platelet count; FIB-4, fibrosis-4 score; APRI, AST-to-platelet ratio index) against mortality (**a**) and mechanical ventilation (**b**).

The performance of each predictor is graphically represented in Figure 1a—ROC curves against mortality, and Figure 1b—ROC curves against mechanical ventilation.

### 3.4. Time-to-Event Analysis

**Figure 2:**
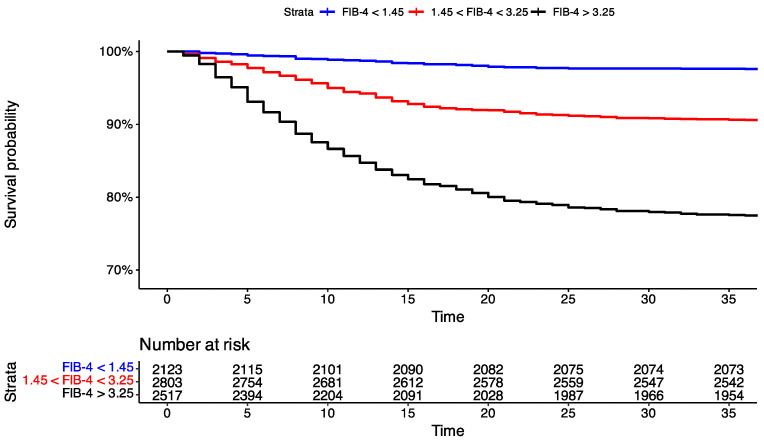
Kaplan–Meier curves of groups stratified with fibrosis-4 score (FIB-4) values.

Additionally, we conducted a time-to-event analysis of mortality using FIB-4 values of the patients who were reported dead (Figure 2). Patients were stratified into three groups based on clinically used ranges of FIB-4. We observed significantly higher mortality among patients with values of FIB-4 above 3.25 and significantly lower mortality among patients with values of FIB-4 below 1.45 (log-rank test < 0.001).

### 3.5. Asymptomatic Patients

Lastly, we present levels of analysed biomarkers and scores among the cohort of asymptomatic patients at the time of admission patients and their demographic and clinical characteristics. Median age in that cohort equalled 65.5 years (IQR: 43–75), and the median BMI = 25.3 kg/m^2^ (IQR: 22.5–28.7). Around half of the patients were male, n = 107 (54.6%). Malignancy was a comorbidity among 30 (15%) patients in that cohort, hypertension among 99 (50.5%) patients, diabetes among 44 (22.4%) patients, ischaemic heart disease among 36 (18.4%) patients, and COPD among 10 (5.1%) patients. Median O_2_ saturation in the studied population equalled 96% (IQR: 95–98); however, 2 (1%) patients later required mechanical ventilation, while 18 (9.2%) patients died during hospitalisation. In asymptomatic patients, hospitalised COVID-19 patients’ cohort median levels of analysed biomarkers at admission equalled to: ALT = 25 IU/L (IQR: 18–44.9), AST = 32 IU/L (IQR: 25–48), GGT = 36 IU/L (IQR: 20–69.3), LDH = 241 IU/L (IQR: 199–323.5), PLT = 203 × 10^3^/μL (IQR: 150–278), APRI = 0.379 (IQR: 0.252–0.67), FIB-4 = 1.836 (IQR: 1.126–3.35), AST/ALT ratio = 1.33 (IQR: 0.972–1.867). These results correspond to 48 (24.5%) patients with ALT values between the upper limit and 2 times the upper limit and 11 (5.6%) patients with values twice as high as the normal range. Fifty-one (26%) patients had AST values between the upper limit and 2 times the normal range, and 16 (8.2%) patients had values that were more than twice as high as the normal range. In addition, there were 64 (32.7%) patients with GGT values above the normal range; 47 (24%) patients with LDH values above the normal range; 49 (25%) patients with PLT counts below the normal range; 20 (10.2%) patients with APRI values above 1.5; 20 (10.2%) patients with APRI values above 1.5; 51 (26%) patients with FIB-4 values above 3.25; and 42 (21.4%) patients with AST/ALT ratio values above 2.

## 4. Discussion

COVID-19 infection can manifest in various ways, from asymptomatic cases to the development of respiratory or multiorgan failure. In patients who develop clinically severe forms, it is important to initiate appropriate treatment. Nonetheless, there remains a need for analysis and detection of factors that would allow us to initiate actions aimed at early detection of patients at risk of requiring mechanical ventilation or at risk of death [13]. In our study, we evaluated levels of selected biomarkers and scores associated with liver function and disease advancement, as well as association of those values with in-hospital mortality and need for mechanical ventilation among hospitalised COVID-19 patients. 

Numerous studies have been conducted to analyse the predictive value of various parameters, such as the presence of specific clinical symptoms, results of morphological tests, levels of cytokines, as well as other laboratory tests, indices, and scales incorporating the selected parameters mentioned above [13,14]. There are many factors associated with an increased risk of either mortality or MV in COVID-19; among those are demographic factors and comorbidities, clinical presentation, and abnormal findings in blood chemistry. Furthermore, researchers evaluated the accuracy of predictive scores used in other infectious diseases, such as CURB-65 used in predicting mortality in community-acquired pneumonia [15]. However, the majority of current data support the notion that the highest accuracy in predicting mortality is achieved using risk scoring systems, specifically developed for COVID-19, such as ISARIC 4C [16]. They provide clinicians with a prognostic tool based on readily available outcomes in hospitalised patients. Research into the changes in biomarker levels on big epidemiological data provides important knowledge on mechanisms of pathogenesis in COVID-19 patients.

In our study, we observed changes in levels of all analysed biomarkers (AST, ALT, GGT, LDH, and PLT) when comparing those who died with those who survived, as well as when comparing those levels in a cohort of patients who required MV with those who did not require MV. Those changes can be attributed to the pathophysiological process that, among many patients, involves liver damage and primarily involves the development of a systemic inflammatory response. One proposed mechanism for such a response is associated with the ability of the SARS-CoV-2 virus to bind to ACE2 receptors on cholangiocytes, which allows the host cell’s endosomal cysteine protease, Cathepsin L, and transmembrane protease serine 2 (TMPRSS2) to cleave the S1 subunit of the spike protein on the virus surface. This leads to viral membrane fusion and subsequent release of viral RNA, enabling replication and destruction of the cells, resulting in cellular dysfunction and potentially triggering a cytokine storm [17,18]. The cytokine storm induces a generalised inflammatory response, damaging multiple organs, including the liver, which results in increased levels of AST, ALT, and GGT and decreased levels of PLT, translating into elevated APRI and FIB-4 indices. The spike protein is also associated with increased clotting by binding to the fibrinogen, a key blood coagulation factor. This interaction promotes the formation of structurally abnormal blood clots with heightened proinflammatory activity. The increased clotting can lead to thrombotic events, which may result in hypoxic damage to the liver tissue [19]. Another potential mechanism of COVID-19 induced liver injury involves neutrophil extracellular traps (NETs), which are released by neutrophils to trap and neutralise pathogens. However, excessive NETs formation can trigger inflammatory responses and activate the coagulation pathway, leading to the formation of blood clots [20]. It is also worth mentioning that medications used in the treatment of COVID-19 can lead to drug-induced liver injury, further contributing to those observations [21].

The results of the multivariate logistic regression models show that an increase in the following biomarkers: AST, ALT, GGT, and LDH, and an increase in the APRI, FIB-4, and AST/ALT ratio scores are all associated with increased odds of mortality, while an increase in PLT count is associated with decreased odds of mortality in the analysed cohort. Our results are consistent with the findings of the meta-analysis by Liu et al., which demonstrated that the FIB-4 index is significantly associated with mortality (relative risk [RR]: 1.47, 95% CI [1.31–1.65], *p* < 0.001, I^2^ = 0%). This study also showed a relationship between the AST/ALT ratio and mortality, with an elevated AST/ALT ratio raising the risk of death by 178% (RR: 2.78, 95% CI [1.10–6.99], *p* = 0.03, I^2^ = 76%) [22]. It is worth noting that the analysis by Liu et al. encompassed nearly the same number of patients as in our research. Similar results were obtained by Raymond et al., where a high FIB-4 score was associated with increased mortality (OR = 3.96, 95% CI [2.16–7.27], *p* < 0.001; I^2^: 41.3%). By comparison, in our study, an increase in the FIB-4 index was associated with increased odds of in-hospital mortality (1.021 [1.002–1.039] per 1 unit change, *p* = 0.024) [23]. Additionally, the results obtained in our analysis for increased odds of mortality with an increase of AST (1.099 [1.075–1.124] per 10 (IU/L) change, *p* < 0.001) are consistent with the results obtained in a large meta-analysis conducted by Del Zompo F, et al., which included approximately 20,000 patients. In this meta-analysis, elevated AST was also associated with increased odds of in-hospital mortality (OR 1.48, 95% CI [1.12–1.96]) [24].

The AST/ALT ratio is not a specific marker for COVID-19, but it may suggest damage to various tissues during the course of a disease caused by SARS-CoV-2. As seen in the results presented above, the increase is more pronounced for AST than ALT. This phenomenon may be due to the fact that ALT is more specific to the liver, so its increase is mainly caused by damage to liver cells. In contrast, AST, which can be associated with the liver, is also common in other tissues such as muscle, skeletal muscle, or kidney. As we know, the damage caused by COVID-19 is multifaceted, so both enzymes increase, but the increase in AST is greater. COVID-19 can lead to various pathological responses, such as cytokine storms, microcirculation damage, and many others. Some of these mechanisms may have a stronger impact on liver cells, leading to an increase in AST levels, but at the same time, ALT levels also rise. An interesting aspect is also the difference in vitamin B6 metabolism, which is required by ALT for its activity. In some cases of COVID-19, decreased pyridoxine consumption may affect ALT activity, leading to a greater increase in AST compared to ALT [25,26].

Although both APRI and FIB-4 scores are used for estimating liver fibrosis, some authors suggest that the FIB-4 marker in COVID-19 infection may not reflect liver fibrosis due to a transient elevation of aminotransferase activity during acute infection, which is used to calculate FIB-4. Consequently, FIB-4 will increase in any condition resulting in an increase in FIB-4 component parameters [27,28]. A similar explanation could be extended to the APRI score. Elevated FIB-4 levels at admission may indicate previously undiagnosed chronic liver disease; however, studies conducted by Kolesova et al., with similar results in the works of Ibáñez-Samaniego et al. and Sterling et al., showed that at admission, FIB-4 was abnormal in an average of 75% of patients with acute conditions at the beginning of treatment, while only 6% of the study group reported previously existing liver disease [27,28,29]. Interestingly, Kolesova et al. and Li et al. demonstrated that the elevation of FIB-4 reflects the severity of COVID-19 associated with liver cell damage linked to systemic inflammation and lung injury during acute infection [29,30]. Furthermore, these two research groups confirmed their results by measuring FIB-4 levels 3–6 months after recovery and found that in a significant proportion of patients, FIB-4 decreased, indicating a relationship between FIB-4 elevation and the level of systemic inflammatory response induced by the COVID-19 disease process [30,31]. As such, COVID-19 infection can significantly decrease the accuracy of those non-invasive tests, which could affect the clinical approach, especially in the case of asymptomatic patients. This view is supported by the fact that 26% of asymptomatic patients in the analysed cohort had values of FIB-4 above 3.25—a threshold suggestive of the presence of fibrosis. The decrease in platelet levels is attributed to the occurrence of coagulopathy and disseminated intravascular coagulation with accompanying high levels of fibrinogen and D-dimers, which are induced by microvascular thrombosis and pulmonary and other organ microcirculation. One of the main reasons for this phenomenon is the heightened immune response triggered by the cytokine storm. However, studies report other pathophysiological mechanisms induced by COVID-19. Specifically, microthrombosis has been observed in organs where the coronavirus was not detected, indicating a mechanism other than just the cytokine storm. This may be associated with other phenomena of severe COVID infection, such as hypoxaemia or ischaemia [32,33,34].

We analysed the predictive value of selected biomarkers and scores against mortality and MV. When analysing the predictive value against mortality, we observed that the FIB-4 index provided the best accuracy in predicting mortality in the analysed cohort, achieving an AUC of 0.73 (95% CI, 0.718–0.749) with an optimal cut-off value equal to 2.764. Similar results were obtained in a study conducted by Sutandyo N. et al., with an AUC of 0.849 (95% CI, 0.735–0.962), *p* < 0.001 [35]. With an optimal cut-off value of FIB-4 equal to 3.85, similar results were also obtained by Sterling et al., with an AUC of 0.77 (95% CI, 0.70–0.84) and Li et al., with an AUC of 0.79; its performance was even better among individuals without a diagnosis of liver disease, with an AUC of 0.85. Our result, based on similar positions in the literature, demonstrates the significant clinical importance of using FIB-4 as a predictor of mortality in patients with COVID-19 [28,30].

The second endpoint of our study was to analyse factors influencing the need for mechanical ventilation in the analysed cohort. We observed increased odds of requiring mechanical ventilation in multivariate logistic regression models among patients with elevated AST, LDH, FIB-4, APRI, and an AST/ALT ratio, as well as a decreased PLT count. Similar research results were obtained by Kabbaha S. et al., who identified several laboratory tests associated with increased risk for invasive mechanical ventilation (IMV), including high AST (OR, 1.71 [95% CI, 1.31–2.22], *p* < 0.001) [36]. Similarly, Ioannou G. et al. found that elevated serum aspartate aminotransferase (>89 U/L vs. ≤25 U/L) was significantly associated with mechanical ventilation (adjusted hazard ratio [aHR], 2.92; 95% CI, [2.13–4.02]) and mortality (aHR, 3.00; 95% CI, [2.21–4.07]) [37].

When comparing the predictive value of analysed parameters, we observed that LDH levels exhibited the highest predictive value for the need for mechanical ventilation in the analysed cohort, with an AUC of 0.753 (95%CI, 0.727–0.778) at the optimal cut-off point of 449.5 IU/L. Similar results for LDH analysis were demonstrated in the study by Li W. et al., with an AUC of 0.757 (95% CI: 0.693–0.823) at the optimal cut-off point of 488 IU/L [38]. Payan-Pernia S. et al. obtained a slightly higher AUC for LDH, with an AUC of 0.891 at the optimal cut-off point of 219 IU/L [39]. Variations in reported AUC values may be attributed to differences in the sample size in the mentioned studies. As we know, lung damage is associated with inflammation induced by the virus. The damage primarily affects lung epithelial cells, resulting in increased LDH levels. The essence of LDH elevation lies in the physiology of cells, where their damage leads to the release of this enzyme in large quantities. The main inflammatory processes associated with COVID affect lung tissue, leading to an increase in LDH. Additionally, this phenomenon may be associated with the liver itself. In the case of direct action of the virus on hepatocytes or tissue responses to increased inflammatory reactions, liver damage may occur, accompanied by an increase in LDH [40,41,42]. Our study showed that aside from LDH, FIB-4 and AST also provide satisfactory predictive value for mechanical ventilation, giving all these factors clinical significance.

The acute course of SARS-CoV-2 infection can lead to significant changes in many commonly analysed markers upon patient admission to the ward. Our study, based on a nationwide database, confirms the important role of liver damage in the course of COVID-19 and provides a basis for making clinical decisions based on analysed markers, with particular emphasis on analysing FIB-4 and LDH to assess the risk of death and mechanical ventilation, respectively. Analysing these specific parameters in COVID-19 patients could be particularly important for initially asymptomatic patients in assessing the risk of further decompensation and the development of a severe course of the disease, which may necessitate mechanical ventilation or lead to patient death.

Lastly, we acknowledge several limitations of our study. Firstly, our results were not validated through a prospective cohort; thus, the predictive value of the study should be interpreted with caution. Secondly, we did not analyse detailed preexisting liver conditions that could affect studied marker levels. Moreover, our results should not be extended when analysing extreme results, as we trimmed the data according to the methodology presented above. Lastly, we did not take into consideration the relation of the analysed markers with other established markers associated with increased odds of mortality and mechanical ventilation.

## 5. Conclusions

To conclude, our research based on a Polish nationwide database of hospitalised COVID-19 patients supports that liver disease advancement and subsequent elevation of specific biomarkers are associated with increased odds of mortality and MV. Moreover, among evaluated factors, FIB-4 presents the highest predictive value against mortality, while LDH levels present the highest predictive value against MV. Thus, such markers are useful in clinical practice. However, these changes could also decrease the accuracy of those estimators in other clinical situations, especially among asymptomatic COVID-19 patients.

## Figures and Tables

**Table 1:** Characterisation of the study population. (IQR, interquartile range; BMI, body mass index; ALT, alanine transaminase; AST, aspartate transaminase; GGT, gamma-glutamyltransferase; LDH, lactate dehydrogenase; PLT, platelet count; FIB-4, fibrosis-4 score; APRI, AST-to-platelet ratio index; COPD, chronic obstructive pulmonary disease).

**Continuous** **Variables**	**Survived** **(n = 6540)** **Median (IQR)**	**Non-Survived** **(n = 904)** **Median (IQR)**	***p*** **Value**	**Non-Ventilated** **(n = 7022)** **Median (IQR)**	**Ventilated** **(n = 422)** **Median (IQR)**	***p*** **Value**
Age, yrs.	63 (46–74)	78 (70–85)	<0.001	65 (48–76)	69 (61–76)	<0.001
BMI, kg/m^2^ (n = 6550)	27.12 (23.8–30.8)	27.03 (23.8–31.1)	0.376	26.99 (23.67–30.67)	28.81 (25.47–32.77)	<0.001
O_2_ saturation (%) (n = 7371)	93 (89–96)	87 (80–90)	<0.001	92 (88–96)	85 (78–89)	<0.001
ALT (IU/L)	29 (19–46)	31 (20.23–50.13)	0.004	28 (19–46)	35.8 (23.7–53.8)	<0.001
AST (IU/L)	38 (27–55)	52 (35–78)	<0.001	38 (27–56)	58 (39.625–78.8)	<0.001
GGT (IU/L) (n = 4891)	39 (22–74)	46 (26–84.95)	<0.001	39 (22–75)	52 (29.8–99)	<0.001
LDH (IU/L) (n = 5835)	321 (238–444)	441 (323.5–597.5)	<0.001	325 (240–448)	489 (380–655)	<0.001
PLT (10^3^/μL)	195 (147–258)	177 (132–236)	<0.001	194 (145–257)	176 (137–227)	<0.001
APRI	0.5 (0.32–0.82)	0.74 (0.45–1.27)	<0.001	0.51 (0.32–0.85)	0.79 (0.52–1.25)	<0.001
FIB-4	2.15(1.2–3.63)	4.02 (2.62–6.63)	<0.001	2.26 (1.27–3.86)	3.61 (2.42–5.19)	<0.001
AST/ALT ratio	1.3 (1–1.77)	1.72 (1.26–2.3)	<0.001	1.33 (1–1.83)	1.61 (1.23–2.08)	<0.001
**Categorical** **Variables**	**Survived** **(n = 6540)** **n (%)**	**Non-Survived** **(n = 904)** **n (%)**	***p*** **Value**	**Non-Ventilated** **(n = 7022)** **n (%)**	**Ventilated** **(n = 422)** **n (%)**	***p*** **Value**
Sex (male)	3458 (52.87)	463 (51.21)	0.368	3674 (52.32)	247 (58.53)	0.015
Malignancy	429 (6.56)	103 (11.39)	<0.001	504 (7.18)	28 (6.64)	0.747
Hypertension	3219 (49.22)	614 (67.92)	<0.001	3564 (50.75)	269 (63.74)	<0.001
Diabetes	1277 (19.52)	320 (35.4)	<0.001	1457 (20.75)	140 (33.18)	<0.001
Ischemic heart disease	787 (12.03	254 (28.1)	<0.001	963 (13.71)	78 (18.48)	0.008
COPD	279 (4.27)	79 (8.7)	<0.001	337 (4.8)	21 (4.98)	0.962

**Table 2:** Univariate and multivariate logistic regression models for the odds of in-hospital death. (OR, odds ratio; CI, confidence interval; ALT, alanine transaminase; AST, aspartate transaminase; GGT, gamma-glutamyltransferase; LDH, lactate dehydrogenase; PLT, platelet count; FIB-4, fibrosis-4 score; APRI, AST-to-platelet ratio index).

Variable	Crude OR (95% CI)	*p* Value	Adjusted OR (95% CI)	*p* Value
ALT (IU/L) per 10 units change	1.02 (0.999–1.041)	0.056	1.033 (1.003–1.063)	0.026
AST (IU/L) per 10 units change	1.109 (1.092–1.126)	<0.001	1.099 (1.075–1.124)	<0.001
GGT (IU/L) per 10 units change	1.011 (1.001–1.021)	0.022	1.015 (1.001–1.028)	0.031
LDH (IU/L) per 10 units change	1.031 (1.027–1.035)	<0.001	1.027 (1.021–1.033)	<0.001
PLT (10^3^/μL) per 10 units	0.979 (0.972–0.987)	<0.001	0.976 (0.966–0.986)	<0.001
APRI per 1 unit change	1.13 (1.085–1.178)	<0.001	1.152 (1.095–1.213)	<0.001
FIB-4 per 1 unit change	1.07 (1.058–1.083)	<0.001	1.038 (1.023–1.053)	<0.001
AST/ALT ratio per 1 unit change	1.778 (1.64–1.928)	<0.001	1.552 (1.393–1.731)	<0.001

**Table 3:** Univariate and multivariate logistic regression models for the odds of mechanical ventilation. (OR, odds ratio; CI, confidence interval; ALT, alanine transaminase; AST, aspartate transaminase; GGT, gamma-glutamyltransferase; LDH, lactate dehydrogenase; PLT, platelet count; FIB-4, fibrosis-4 score; APRI, AST-to-platelet ratio index).

Variable	Crude OR (95% CI)	*p* Value	Adjusted OR (95% CI)	*p* Value
ALT (IU/L) per 10 units change	1.05 (1.023–1.077)	<0.001	1.009 (0.972–1.045)	0.615
AST (IU/L) per 10 units change	1.103 (1.082–1.124)	<0.001	1.071 (1.043–1.098)	<0.001
GGT (IU/L) per 10 units change	1.013 (0.999–1.026)	0.065	1.014 (0.993–1.03)	0.126
LDH (IU/L) per 10 units change	1.038 (1.033–1.043)	<0.001	1.035 (1.035–1.026)	<0.001
PLT (10^3^/μL) per 10 units	0.982 (0.971–0.993)	0.002	0.974 (0.961–0.987)	<0.001
APRI per 1 unit change	1.11 (1.056–1.163)	<0.001	1.092 (1.028–1.153)	0.002
FIB-4 per 1 unit change	1.035 (1.02–1.049)	<0.001	1.021 (1.002–1.039)	0.024
AST/ALT ratio per 1 unit change	1.402 (1.268–1.547)	<0.001	1.461 (1.285–1.658)	<0.001

**Table 4:** Receiver operator characteristic (ROC) curve analysis against mortality. (AUC, area under the curve; CI, confidence interval; FPR, false positive rate; ALT, alanine transaminase; AST, aspartate transaminase; GGT, gamma-glutamyltransferase; LDH, lactate dehydrogenase; PLT, platelet count; FIB-4, fibrosis-4 score; APRI, AST-to-platelet ratio index).

Variable	AUC	95% CI	Optimal Cut-Off Point	Sensitivity at Optimal Cut-Off Point	FPR at Optimal Cut-Off
FIB-4	0.733	0.718–0.749	2.764	0.7289823	0.3681957
LDH (IU/L) (n = 5835)	0.681	0.659–0.702	359.5	0.680315	0.4094231
AST/ALT ratio	0.654	0.634–0.673	1.566	0.5951327	0.3457187
AST (IU/L)	0.649	0.629–0.668	46.05	0.5873894	0.3498471
APRI	0.641	0.622–0.66	0.616	0.6073009	0.3818043
PLT (10^3^/μL)	0.563	0.543–0.583	170.5	0.4734513	0.3718654
GGT (IU/L) (n = 4891)	0.55	0.526–0.574	44.4	0.5234114	0.4411833
ALT (IU/L)	0.529	0.509–0.549	29.15	0.5365044	0.4824159

**Table 5:** Receiver operator characteristic (ROC) curve analysis against mechanical ventilation. (AUC, area under the curve; CI, confidence interval; FPR, false positive rate; ALT, alanine transaminase; AST, aspartate transaminase; GGT, gamma-glutamyltransferase; LDH, lactate dehydrogenase; PLT, platelet count; FIB-4, fibrosis-4 score; APRI, AST-to-platelet ratio index).

Variable	AUC	95% CI	Optimal Cut-Off Point	Sensitivity at Optimal Cut-Off Point	FPR at Optimal Cut-Off
LDH (IU/L) (n = 5835)	0.753	0.727–0.778	449.5	0.6389776	0.2480985
AST (IU/L)	0.69	0.666–0.715	46.15	0.6635071	0.3614355
FIB-4	0.673	0.65–0.695	2.544	0.7322275	0.4400456
APRI	0.663	0.638–0.687	0.616	0.6658768	0.3937625
AST/ALT ratio	0.609	0.582–0.639	1.23	0.7535545	0.7535545
ALT (IU/L)	0.591	0.564–0.617	27.65	0.6729858	0.5206494
GGT (IU/L) (n = 4891)	0.59	0.558–0.622	40.65	0.6425993	0.479844
PLT (10^3^/μL)	0.558	0.531–0.585	193.5	0.6184834	0.4998576

## Data Availability

Supporting reported results can be provided upon request from the corresponding author.

## Supplementary Materials

The following supporting information can be downloaded at: https://www.mdpi.com/article/10.3390/v16101530/s1. Table S1: Multivariate logistic regression models for the odds of in-hospital death. (AIC, Akaike information criterion; OR, odds ratio; CI, confidence interval; BMI, body mass index; ALT, alanine transaminase; AST, aspartate transaminase; GGT, gamma-glutamyltransferase; LDH, lactate dehydrogenase; PLT, platelet count; FIB-4, fibrosis-4 score; APRI, AST-to-platelet ratio index); Table S2: Multivariate logistic regression models for the odds of mechanical ventilation. (AIC, Akaike information criterion; OR, odds ratio; CI, confidence interval; BMI, body mass index; ALT, alanine transaminase; AST, aspartate transaminase; GGT, gamma-glutamyltransferase; LDH, lactate dehydrogenase; PLT, platelet count; FIB-4, fibrosis-4 score; APRI, AST-to-platelet ratio index).

## References

[1] Yüce M., Filiztekin E., Özkaya K.G. (2021). COVID-19 Diagnosis—A Review of Current Methods. Biosens. Bioelectron..

[2] Nardo A.D., Schneeweiss-Gleixner M., Bakail M., Dixon E.D., Lax S.F., Trauner M. (2021). Pathophysiological Mechanisms of Liver Injury in COVID-19. Liver Int..

[3] Dufour J.-F., Marjot T., Becchetti C., Tilg H. (2022). COVID-19 and Liver Disease. Gut.

[4] Agrawal S., Dhiman R.K., Limdi J.K. (2016). Evaluation of Abnormal Liver Function Tests. Postgrad. Med. J..

[5] Read J.A., Winter V.J., Eszes C.M., Sessions R.B., Brady R.L. (2001). Structural Basis for Altered Activity of M- and H-Isozyme Forms of Human Lactate Dehydrogenase. Proteins.

[6] Shokri Afra H., Amiri-Dashatan N., Ghorbani F., Maleki I., Rezaei-Tavirani M. (2020). Positive Association between Severity of COVID-19 Infection and Liver Damage: A Systematic Review and Meta-Analysis. Gastroenterol. Hepatol. Bed Bench.

[7] Barrett T.J., Bilaloglu S., Cornwell M., Burgess H.M., Virginio V.W., Drenkova K., Ibrahim H., Yuriditsky E., Aphinyanaphongs Y., Lifshitz M. (2021). Platelets Contribute to Disease Severity in COVID-19. J. Thromb. Haemost..

[8] McConnell M.J., Kondo R., Kawaguchi N., Iwakiri Y. (2022). COVID-19 and Liver Injury: Role of Inflammatory Endotheliopathy, Platelet Dysfunction, and Thrombosis. Hepatol. Commun..

[9] Xu X., Jiang L., Wu C., Pan L., Lou Z., Peng C., Dong Y., Ruan B. (2022). The Role of Fibrosis Index FIB-4 in Predicting Liver Fibrosis Stage and Clinical Prognosis: A Diagnostic or Screening Tool?. J. Formos. Med. Assoc..

[10] Zhang J., Liu F., Song T., Li Z., Xia P., Tang X., Xu M., Shen Y., Ma J., Liu X. (2022). Liver Fibrosis Scores and Clinical Outcomes in Patients with COVID-19. Front. Med..

[11] Jaroszewicz J., Kowalska J., Pawłowska M., Rogalska M., Zarębska-Michaluk D., Rorat M., Lorenc B., Czupryna P., Sikorska K., Piekarska A. (2022). Remdesivir Decreases Mortality in COVID-19 Patients with Active Malignancy. Cancers.

[12] DeLong E.R., DeLong D.M., Clarke-Pearson D.L. (1988). Comparing the Areas under Two or More Correlated Receiver Operating Characteristic Curves: A Nonparametric Approach. Biometrics.

[13] Elshazli R.M., Toraih E.A., Elgaml A., El-Mowafy M., El-Mesery M., Amin M.N., Hussein M.H., Killackey M.T., Fawzy M.S., Kandil E. (2020). Diagnostic and Prognostic Value of Hematological and Immunological Markers in COVID-19 Infection: A Meta-Analysis of 6320 Patients. PLoS ONE.

[14] Zhang H., Wu H., Pan D., Shen W. (2022). D-Dimer Levels and Characteristics of Lymphocyte Subsets, Cytokine Profiles in Peripheral Blood of Patients with Severe COVID-19: A Systematic Review and Meta-Analysis. Front. Med..

[15] Bradley J., Sbaih N., Chandler T.R., Furmanek S., Ramirez J.A., Cavallazzi R. (2022). Pneumonia Severity Index and CURB-65 Score Are Good Predictors of Mortality in Hospitalized Patients with SARS-CoV-2 Community-Acquired Pneumonia. Chest.

[16] Gupta R.K., Harrison E.M., Ho A., Docherty A.B., Knight S.R., van Smeden M., Abubakar I., Lipman M., Quartagno M., Pius R. (2021). Development and Validation of the ISARIC 4C Deterioration Model for Adults Hospitalised with COVID-19: A Prospective Cohort Study. Lancet Respir. Med..

[17] Yuki K., Fujiogi M., Koutsogiannaki S. (2020). COVID-19 Pathophysiology: A Review. Clin. Immunol..

[18] Liu T., Luo S., Libby P., Shi G.-P. (2020). Cathepsin L-Selective Inhibitors: A Potentially Promising Treatment for COVID-19 Patients. Pharmacol. Ther..

[19] Ryu J.K., Sozmen E.G., Dixit K., Montano M., Matsui Y., Liu Y., Helmy E., Deerinck T.J., Yan Z., Schuck R. (2021). SARS-CoV-2 Spike Protein Induces Abnormal Inflammatory Blood Clots Neutralized by Fibrin Immunotherapy. Biorxiv.

[20] Li D., Ding X., Xie M., Tian D., Xia L. (2021). COVID-19-Associated Liver Injury: From Bedside to Bench. J. Gastroenterol..

[21] Ma C., Cong Y., Zhang H. (2020). COVID-19 and the Digestive System. Am. J. Gastroenterol..

[22] Liu M., Mei K., Tan Z., Huang S., Liu F., Deng C., Ma J., Yu P., Liu X. (2022). Liver Fibrosis Scores and Hospitalization, Mechanical Ventilation, Severity, and Death in Patients with COVID-19: A Systematic Review and Dose-Response Meta-Analysis. Can. J. Gastroenterol. Hepatol..

[23] Pranata R., Yonas E., Huang I., Lim M.A., Nasution S.A., Kuswardhani R.A.T. (2021). Fibrosis-4 Index and Mortality in Coronavirus Disease 2019: A Meta-Analysis. Eur. J. Gastroenterol. Hepatol..

[24] Del Zompo F., De Siena M., Ianiro G., Gasbarrini A., Pompili M., Ponziani F.R. (2020). Prevalence of Liver Injury and Correlation with Clinical Outcomes in Patients with COVID-19: Systematic Review with Meta-Analysis. Eur. Rev. Med. Pharmacol. Sci..

[25] Cha M.H., Regueiro M., Sandhu D.S. (2020). Gastrointestinal and Hepatic Manifestations of COVID-19: A Comprehensive Review. World J. Gastroenterol..

[26] Zaim S., Chong J.H., Sankaranarayanan V., Harky A. (2020). COVID-19 and Multiorgan Response. Curr. Probl. Cardiol..

[27] Ibáñez-Samaniego L., Bighelli F., Usón C., Caravaca C., Fernández Carrillo C., Romero M., Barreales M., Perelló C., Madejón A., Marcos A.C. (2020). Elevation of Liver Fibrosis Index FIB-4 Is Associated with Poor Clinical Outcomes in Patients with COVID-19. J. Infect. Dis..

[28] Sterling R.K., Oakes T., Gal T.S., Stevens M.P., deWit M., Sanyal A.J. (2020). The Fibrosis-4 Index Is Associated with Need for Mechanical Ventilation and 30-Day Mortality in Patients Admitted with Coronavirus Disease 2019. J. Infect. Dis..

[29] Kolesova O., Vanaga I., Laivacuma S., Derovs A., Kolesovs A., Radzina M., Platkajis A., Eglite J., Hagina E., Arutjunana S. (2021). Intriguing Findings of Liver Fibrosis Following COVID-19. BMC Gastroenterol..

[30] Li Y., Regan J., Fajnzylber J., Coxen K., Corry H., Wong C., Rosenthal A., Atyeo C., Fischinger S., Gillespie E. (2021). Liver Fibrosis Index FIB-4 Is Associated with Mortality in COVID-19. Hepatol. Commun..

[31] Henry B.M., de Oliveira M.H.S., Benoit S., Plebani M., Lippi G. (2020). Hematologic, Biochemical and Immune Biomarker Abnormalities Associated with Severe Illness and Mortality in Coronavirus Disease 2019 (COVID-19): A Meta-Analysis. Clin. Chem. Lab. Med..

[32] Zhang T., Sun L.X., Feng R.E. (2020). Comparison of clinical and pathological features between severe acute respiratory syndrome and coronavirus disease 2019. Zhonghua Jie He He Hu Xi Za Zhi.

[33] Sivaloganathan H., Ladikou E.E., Chevassut T. (2020). COVID-19 Mortality in Patients on Anticoagulants and Antiplatelet Agents. Br. J. Haematol..

[34] Cui S., Chen S., Li X., Liu S., Wang F. (2020). Prevalence of Venous Thromboembolism in Patients with Severe Novel Coronavirus Pneumonia. J. Thromb. Haemost..

[35] Sutandyo N., Kurniawati S.A., Jayusman A.M., Syafiyah A.H., Pranata R., Hanafi A.R. (2021). Repurposing FIB-4 Index as a Predictor of Mortality in Patients with Hematological Malignancies and COVID-19. PLoS ONE.

[36] Kabbaha S., Al-Azzam S., Karasneh R.A., Khassawneh B.Y., Al-Mistarehi A.-H., Lattyak W.J., Aldiab M., Hasan S.S., Conway B.R., Aldeyab M.A. (2022). Predictors of Invasive Mechanical Ventilation in Hospitalized COVID-19 Patients: A Retrospective Study from Jordan. Expert Rev. Respir. Med..

[37] Ioannou G.N., Locke E., Green P., Berry K., O’Hare A.M., Shah J.A., Crothers K., Eastment M.C., Dominitz J.A., Fan V.S. (2020). Risk Factors for Hospitalization, Mechanical Ventilation, or Death Among 10 131 US Veterans with SARS-CoV-2 Infection. JAMA Netw. Open.

[38] Li W., Lin F., Dai M., Chen L., Han D., Cui Y., Pan P. (2020). Early Predictors for Mechanical Ventilation in COVID-19 Patients. Ther. Adv. Respir. Dis..

[39] Payán-Pernía S., Gómez Pérez L., Remacha Sevilla Á.F., Sierra Gil J., Novelli Canales S. (2021). Absolute Lymphocytes, Ferritin, C-Reactive Protein, and Lactate Dehydrogenase Predict Early Invasive Ventilation in Patients with COVID-19. Lab. Med..

[40] Mihai N., Lazar M., Tiliscan C., Barbu E.C., Chitu C.E., Stratan L., Ganea O.A., Arama S.S., Ion D.A., Arama V. (2022). Predictors of Liver Injury in Hospitalized Patients with SARS-CoV-2 Infection. Medicina.

[41] Luo M., Ballester M.P., Soffientini U., Jalan R., Mehta G. (2022). SARS-CoV-2 Infection and Liver Involvement. Hepatol. Int..

[42] Uzum Y., Turkkan E., Uzum Y., Turkkan E. (2023). Predictivity of CRP, Albumin, and CRP to Albumin Ratio on the Development of Intensive Care Requirement, Mortality, and Disease Severity in COVID-19. Cureus.

