# Supplementary material for: Association between Liver Damage and Disease Progression Markers with Mortality Risk and Mechanical Ventilation in Hospitalized COVID-19 Patients: A Nationwide Retrospective SARSTer Study
Source: Viruses. 2024 Sep 27;16(10):1530. doi: 10.3390/v16101530 (PMC11512261; doi:10.3390/v16101530)
Supplement: Supplementary file 1 [file viruses-16-01530-s001.zip › viruses-3179327-supplementary.pdf]

Table S1. Multivariate logistic regression models for the odds of in-hospital death. (AIC, Akaike information criterion; OR, odds ratio; CI, confidence interval; BMI, body mass index; ALT, alanine transaminase; AST, aspartate transaminase; GGT, gamma-glutamyltransferase; LDH, lactate dehydrogenase; PLT, platelet count; FIB-4, fibrosis-4 score; APRI, AST-to-platelet ratio index).

Variable	Adjusted OR (95% CI)	*p* value
	AIC: 3186.2	
Intercept	131.384 (28.727 - 609.442)	<0.001
Age (years) per 1 unit change	1.069 (1.06 - 1.078)	<0.001
Sex (male)	1.224 (1.014 - 1.478)	0.035
BMI per 1 unit change	0.984 (0.965 - 1.002)	0.08
O_2 saturation at the admission (%) per 1 unit change	0.876 (0.864 - 0.888)	<0.001
ALT (IU/l) per 10 units change	1.033 (1.003 - 1.063)	0.026
Malignancy	1.392 (1.031 - 1.86)	0.028
Hypertension	1.031 (0.833 - 1.278)	0.78
Diabetes	1.628 (1.33 - 1.99)	<0.001
Ischemic heart disease	1.455 (1.172 - 1.801)	0.001
COPD	1.013 (0.724 - 1.399)	0.939
	AIC: 3123.5	
Intercept	56.29 (12.219 - 262.385)	<0.001
Age (years) per 1 unit change	1.069 (1.06 - 1.078)	<0.001
Sex (male)	1.182 (0.978 - 1.43)	0.084
BMI per 1 unit change	0.983 (0.964 - 1.001)	0.067
O_2 saturation at the admission (%) per 1 unit change	0.881 (0.869 - 0.893)	<0.001
AST (IU/l) per 10 units change	1.099 (1.075 - 1.124)	<0.001
Malignancy	1.476 (1.091 - 1.977)	0.01
Hypertension	1.04 (0.839 - 1.292)	0.723
Diabetes	1.693 (1.38 - 2.074)	<0.001
Ischemic heart disease	1.439 (1.156 - 1.786)	0.001
COPD	1.071 (0.763 - 1.485)	0.685
	AIC: 2245	
Intercept	107.278 (17.629 - 664.911)	<0.001
Age (years) per 1 unit change	1.067 (1.057 - 1.078)	<0.001
Sex (male)	1.24 (0.991 - 1.554)	0.06
BMI per 1 unit change	0.978 (0.956 - 1)	0.053
O_2 saturation at the admission (%) per 1 unit change	0.882 (0.868 - 0.896)	<0.001
GGT (IU/l) per 10 units change	1.015 (1.001 - 1.028)	0.031
Malignancy	1.35 (0.955 - 1.882)	0.082
Hypertension	0.947 (0.738 - 1.217)	0.667
Diabetes	1.442 (1.129 - 1.838)	0.003
Ischemic heart disease	1.217 (0.934 - 1.579)	0.143

COPD	0.898 (0.588 - 1.338)	0.607
AIC: 2379.2		
Intercept	3.646 (0.537 - 24.828)	0.186
Age (years) per 1 unit change	1.074 (1.064 - 1.086)	<0.001
Sex (male)	1.111 (0.893 - 1.382)	0.347
BMI per 1 unit change	0.983 (0.962 - 1.004)	0.123
O_2 saturation at the admission (%) per 1 unit change	0.897 (0.883 - 0.912)	<0.001
LDH (IU/l) per 10 units change	1.027 (1.021 - 1.033)	<0.001
Malignancy	1.527 (1.089 - 2.114)	0.012
Hypertension	1.126 (0.88 - 1.446)	0.348
Diabetes	1.637 (1.292 - 2.072)	<0.001
Ischemic heart disease	1.306 (1.005 - 1.689)	0.043
COPD	1.048 (0.701 - 1.534)	0.816
AIC: 3170.3		
Intercept	505.926 (105.573 - 2468.265)	<0.001
Age (years) per 1 unit change	1.066 (1.057 - 1.075)	<0.001
Sex (male)	1.207 (1 - 1.458)	0.05
BMI per 1 unit change	0.982 (0.964 - 1)	0.059
O_2 saturation at the admission (%) per 1 unit change	0.871 (0.859 - 0.883)	<0.001
PLT (10^3/μl) per 10 units	0.976 (0.966 - 0.986)	<0.001
Malignancy	1.374 (1.017 - 1.837)	0.035
Hypertension	1.045 (0.844 - 1.296)	0.688
Diabetes	1.654 (1.351 - 2.024)	<0.001
Ischemic heart disease	1.491 (1.201 - 1.847)	<0.001
COPD	0.989 (0.707 - 1.365)	0.945
AIC: 3164		
Intercept	153.448 (34.223 - 697.933)	<0.001
Age (years) per 1 unit change	1.069 (1.06 - 1.078)	<0.001
Sex (male)	1.228 (1.018 - 1.482)	0.032
BMI per 1 unit change	0.984 (0.965 - 1.002)	0.081
O_2 saturation at the admission (%) per 1 unit change	0.874 (0.862 - 0.886)	<0.001
APRI per 1 unit change	1.152 (1.095 - 1.213)	<0.001
Malignancy	1.411 (1.045 - 1.886)	0.022
Hypertension	1.04 (0.84 - 1.29)	0.721
Diabetes	1.635 (1.335 - 2)	<0.001
Ischemic heart disease	1.49 (1.2 - 1.845)	<0.001
COPD	1.022 (0.73 - 1.412)	0.895
AIC: 3167.1		
Intercept	205.791 (45.857 - 937.412)	<0.001
Age (years) per 1 unit change	1.064 (1.056 - 1.074)	<0.001

Sex (male)	1.23 (1.019 - 1.484)	0.031
BMI per 1 unit change	0.984 (0.966 - 1.002)	0.083
O_2 saturation at the admission (%) per 1 unit change	0.874 (0.862 - 0.886)	<0.001
FIB-4 per 1 unit change	1.038 (1.023 - 1.053)	<0.001
Malignancy	1.413 (1.046 - 1.888)	0.022
Hypertension	1.039 (0.84 - 1.29)	0.724
Diabetes	1.641 (1.34 - 2.008)	<0.001
Ischemic heart disease	1.502 (1.21 - 1.862)	<0.001
COPD	1.013 (0.724 - 1.398)	0.94
AIC: 3125.5		
Intercept	93.445 (20.753 - 426.261)	<0.001
Age (years) per 1 unit change	1.061 (1.052 - 1.07)	<0.001
Sex (male)	1.307 (1.081 - 1.581)	0.006
BMI per 1 unit change	0.989 (0.971 - 1.007)	0.223
O_2 saturation at the admission (%) per 1 unit change	0.877 (0.865 - 0.889)	<0.001
AST / ALT ratio per 1 unit change	1.552 (1.393 - 1.731)	<0.001
Malignancy	1.407 (1.04 - 1.883)	0.024
Hypertension	1.019 (0.821 - 1.266)	0.868
Diabetes	1.738 (1.417 - 2.131)	<0.001
Ischemic heart disease	1.464 (1.177 - 1.819)	0.001
COPD	0.991 (0.706 - 1.372)	0.956

Table S2. Multivariate logistic regression models for the odds of mechanical ventilation. (AIC, Akaike information criterion; OR, odds ratio; CI, confidence interval; BMI, body mass index; ALT, alanine transaminase; AST, aspartate transaminase; GGT, gamma-glutamyltransferase; LDH, lactate dehydrogenase; PLT, platelet count; FIB-4, fibrosis-4 score; APRI, AST-to-platelet ratio index).

Variable	Adjusted OR (95% CI)	*p* value
AIC: 2275.3		
Intercept	401.527 (77.152 - 2106.06)	<0.001
Age (years) per 1 unit change	1.008 (0.999 - 1.016)	0.079
Sex (male)	1.311 (1.029 - 1.674)	0.029
BMI per 1 unit change	1.031 (1.011 - 1.051)	0.002
O_2 saturation at the admission (%) per 1 unit change	0.887 (0.874 - 0.9)	<0.001
ALT (IU/l) per 10 units change	1.009 (0.972 - 1.045)	0.615
Malignancy	0.898 (0.551 - 1.395)	0.648
Hypertension	1.184 (0.901 - 1.561)	0.228
Diabetes	1.372 (1.046 - 1.792)	0.021
Ischemic heart disease	1.059 (0.764 - 1.451)	0.726
COPD	0.592 (0.332 - 0.99)	0.058
AIC: 2252.1		
Intercept	198.138 (37.779 - 1046.451)	<0.001
Age (years) per 1 unit change	1.007 (0.999 - 1.016)	0.089
Sex (male)	1.244 (0.977 - 1.588)	0.078
BMI per 1 unit change	1.03 (1.01 - 1.05)	0.002
O_2 saturation at the admission (%) per 1 unit change	0.891 (0.878 - 0.904)	<0.001
AST (IU/l) per 10 units change	1.071 (1.043 - 1.098)	<0.001
Malignancy	0.943 (0.578 - 1.466)	0.802
Hypertension	1.19 (0.906 - 1.569)	0.215
Diabetes	1.409 (1.074 - 1.843)	0.013
Ischemic heart disease	1.05 (0.756 - 1.442)	0.765
COPD	0.616 (0.345 - 1.033)	0.081
AIC: 1554.9		
Intercept	711.757 (93.323 - 5473.423)	<0.001
Age (years) per 1 unit change	1.001 (0.991 - 1.012)	0.815
Sex (male)	1.149 (0.857 - 1.545)	0.355
BMI per 1 unit change	1.031 (1.007 - 1.055)	0.009
O_2 saturation at the admission (%) per 1 unit change	0.887 (0.872 - 0.903)	<0.001
GGT (IU/l) per 10 units change	1.014 (0.993 - 1.03)	0.126
Malignancy	0.819 (0.452 - 1.386)	0.483
Hypertension	1.045 (0.755 - 1.454)	0.791
Diabetes	1.195 (0.851 - 1.664)	0.296

Ischemic heart disease	1.059 (0.701 - 1.569)	0.779
COPD	0.748 (0.377 - 1.364)	0.374
AIC: 1765.1		
Intercept	17.044 (2.139 - 134.919)	0.007
Age (years) per 1 unit change	1.01 (1 - 1.02)	0.063
Sex (male)	1.12 (0.853 - 1.475)	0.415
BMI per 1 unit change	1.023 (0.999 - 1.046)	0.05
O_2 saturation at the admission (%) per 1 unit change	0.909 (0.893 - 0.925)	<0.001
LDH (IU/l) per 10 units change	1.035 (1.035 - 1.026)	<0.001
Malignancy	1.065 (0.641 - 1.692)	0.798
Hypertension	1.289 (0.95 - 1.757)	0.106
Diabetes	1.331 (0.976 - 1.804)	0.068
Ischemic heart disease	1.161 (0.794 - 1.67)	0.429
COPD	0.667 (0.344 - 1.197)	0.2
AIC: 2260.1		
Intercept	1262.322 (225.382 - 7180.603)	<0.001
Age (years) per 1 unit change	1.005 (0.997 - 1.014)	0.219
Sex (male)	1.266 (0.994 - 1.616)	0.057
BMI per 1 unit change	1.03 (1.01 - 1.05)	0.003
O_2 saturation at the admission (%) per 1 unit change	0.883 (0.87 - 0.896)	<0.001
PLT (10^3/μl) per 10 units	0.974 (0.961 - 0.987)	<0.001
Malignancy	0.878 (0.538 - 1.366)	0.583
Hypertension	1.198 (0.912 - 1.581)	0.197
Diabetes	1.389 (1.058 - 1.816)	0.017
Ischemic heart disease	1.078 (0.777 - 1.478)	0.647
COPD	0.6 (0.338 - 1.002)	0.064
AIC: 2267.9		
Intercept	390.783 (76.638 - 2010.434)	<0.001
Age (years) per 1 unit change	1.007 (0.999 - 1.016)	0.093
Sex (male)	1.303 (1.025 - 1.661)	0.032
BMI per 1 unit change	1.031 (1.012 - 1.051)	0.002
O_2 saturation at the admission (%) per 1 unit change	0.887 (0.874 - 0.9)	<0.001
APRI per 1 unit change	1.092 (1.028 - 1.153)	0.002
Malignancy	0.909 (0.558 - 1.412)	0.685
Hypertension	1.187 (0.904 - 1.566)	0.22
Diabetes	1.375 (1.048 - 1.796)	0.021
Ischemic heart disease	1.077 (0.776 - 1.477)	0.649
COPD	0.601 (0.337 - 1.005)	0.066
AIC: 2271.1		
Intercept	434.763 (85.502 - 2230.983)	<0.001

Age (years) per 1 unit change	1.005 (0.997 - 1.014)	0.207
Sex (male)	1.31 (1.03 - 1.67)	0.028
BMI per 1 unit change	1.032 (1.012 - 1.052)	0.001
O_2 saturation at the admission (%) per 1 unit change	0.887 (0.874 - 0.899)	<0.001
FIB-4 per 1 unit change	1.021 (1.002 - 1.039)	0.024
Malignancy	0.908 (0.557 - 1.412)	0.683
Hypertension	1.19 (0.906 - 1.57)	0.214
Diabetes	1.379 (1.051 - 1.802)	0.019
Ischemic heart disease	1.073 (0.773 - 1.471)	0.667
COPD	0.598 (0.336 - 1.001)	0.064
AIC: 2242.6		
Intercept	193.882 (37.525 - 1008.938)	<0.001
Age (years) per 1 unit change	1.004 (0.996 - 1.013)	0.284
Sex (male)	1.375 (1.08 - 1.756)	0.01
BMI per 1 unit change	1.037 (1.018 - 1.057)	<0.001
O_2 saturation at the admission (%) per 1 unit change	0.889 (0.875 - 0.902)	<0.001
AST / ALT ratio per 1 unit change	1.461 (1.285 - 1.658)	<0.001
Malignancy	0.92 (0.564 - 1.433)	0.725
Hypertension	1.173 (0.891 - 1.55)	0.26
Diabetes	1.441 (1.096 - 1.887)	0.008
Ischemic heart disease	1.016 (0.73 - 1.396)	0.924
COPD	0.582 (0.326 - 0.977)	0.052
